# The Psychosocial Fuzziness of Fear in the Coronavirus (COVID-19) Era and the Role of Robots

**DOI:** 10.3389/fpsyg.2020.02245

**Published:** 2020-09-24

**Authors:** Antonella Marchetti, Cinzia Di Dio, Davide Massaro, Federico Manzi

**Affiliations:** Research Unit on Theory of Mind, Department of Psychology, Catholic University of the Sacred Heart, Milan, Italy

**Keywords:** coronavirus, COVID-19, fear, social distancing, close relationships, robots

The coronavirus disease 2019 (COVID-19) pandemic has prompted much research on the possible use of robots in different areas of intervention. One of them is related to the deployment of social robots to cope with different needs elicited by and depending on the emergency. According to a recent article published in *Science* (Yang et al., 2020, p. 1) “social robots could be deployed to provide continued social interactions and adherence to treatment regimens without *fear* of spreading disease.” In this context, social isolation and quarantine—often significantly prolonged due to the duration of the infection—have plausibly exerted a negative impact on well-being and perhaps mental health, whose jeopardy was even more likely if a previous psychological vulnerability was present. If historically robots have been employed in dangerous and risky duties, presently, some of the most promising domains of robots' development also include rehabilitation, caring, and educational and clinic intervention. We are witnessing a shift from the concept of “robot as slaves” to “robots as companions, nurses, teachers…” that, in a word, behave, interact, and work “like us” (cfr. Marchetti et al., 2018). Yang et al. argue that social robots used to “adherence to treatment regimens without spreading of fear” need to be implemented following sophisticated human models, including mental states like emotions and beliefs, as well as the context and environment of the interaction (p. 2). In our opinion, the “environments” are the affordances strictly linked to survival in an evolutionary sense. The “context” is represented by everyday life socio-material and socio-cognitive cues. Furthermore, we believe that the implementation of social robots based on every possible human model cannot merely be the product of “a fusion of engineering and infectious disease professionals” (Yang et al., 2020, p. 2). The model would require an interdisciplinary perspective that includes also the contribution of psychologists. The recent pandemic has in fact laid the foundations for rereading our daily relationships from the point of view of not only human relations but also other agents, such as robots. In the present Opinion, we therefore suggest that the use of robots is not only a purely technical issue but also supported by important changes in the way we view relationships, particularly with those who are close to us. With this aim in mind, we focused on identifying some psychological components most subject to change due to the current global situation. Let's take, for example, the emotion of fear mentioned above. Fear will probably take (if not already has) a different form because of the virus. Fear is a primary (Ekman and Friesen, 1971) and adaptive emotion developed through evolution to enable coping with danger and ensure survival. Predators, contaminants, and invaders are the potentially dangerous enemies that are all risky variables toward which close relationships usually act as protective factors. In case of fear, the options for the individual are represented by the so-called “fight or flight” behaviors. On the relational level, it is the search for a secure base (Bowlby, 1988), where a place can be found for reassurance and affective supply. This tendency persists also in adulthood due to the transgenerational transmission of attachment patterns.

Nonetheless, COVID-19 pandemic confronted us with a scenario where “fear has no face.” Now, it also involves close relationship partners, i.e., people who potentially are sources or recipients of care. This profoundly contrasts with a series of fundamental developmental achievements that make physical proximity the embodied prototype of psychological proximity. The individual undertakes a path in which the “known social other”/“unknown social other” dichotomy acts as an organizer of beliefs and attitudes, thus contributing to the construction of the Self as a distinct and separate entity from the Other. From a sensorineural point of view, the human baby is equipped to recognize and trustfully orient herself/himself toward primary figures of care and protection; it is precisely on this basis that trust is built in others and ourselves (Di Dio et al., 2019, 2020a,b; Manzi et al., 2020a,b). The so-called “anguish of the stranger” (Spitz, 1945; Schaffer, 1966) emerges around 8 months of age. It marks the distinction between the caregivers and all the others: before becoming a neutral agent that the child will observe and know, the “other” *per se* is perceived as scary (worthy of fear in other words). This step appears to be in line with the older child's behavior observed within the Strange Situation (a paradigm aimed at evaluating attachment; Ainsworth et al., 1978): the response of distress and fear toward the stranger, who is generally more accepted if the mother is at the child's presence, and the reactions toward whom are predicted by the security of the child's attachment to the mother. Later in life, the developing child can establish attachment bonds with other people in her/his life contexts: friends, schoolmates, relatives of the extended family, teachers, and educators in various contexts, from school to sports activities (Pianta, 1999). While the theoretical perspective of multiple attachments postulates that the widening of the “known social other” sphere is characterized by a differentiation of the functional roles played by multiple relationships, it maintains the fundamental developmental ability to identify the other as a “secure-safe social partner,” distinguishing him/her from the “risky-unsafe social partner.” The possibility to create multiple attachments prevents a series of developmental risks and acts as an enhancer of positive primary attachment relationships and as a vicarious protective factor in the conditions of relational affective fragility. Besides, not only are secure relationships with multiple figures—with the teacher, just to give an example—connected with the personal well-being within the affective sphere, but also with cognitive performance at school, as well as with socio-cognitive indexes like school climate, peer acceptance, and so on. In order to exert an enhancing-protective role, all these “others” (educators, teachers, relatives) have to be perceived as “besides me.” The physical sense of “besides” —in its literal meaning—anticipates in development, and continues to support in the life span, the metaphorical sense of the human experience of psychological closeness and proximity. And it is precisely the impossibility to fully get the chances offered by the different meanings of “besideness” (physical proximity and security/safeness) that is responsible for the erosion of the feeling of being protected from fear within the contexts of affective bonds. Although technology allows us to be connected even when physically separated, the experienced loneliness and isolation largely reported during COVID-19 may depend both on the technological inability to embody affective relationships and perhaps also on more or less implicit awareness that “the known social other” (also my caregiver, daughter-son-teacher-girlfriend/boyfriend-teacher, educator) could be dangerous for me. Consequently, the pervasive mood of close relationships is no longer that of security but rather a widespread sense of fuzzy fear (Furthermore, if people reflect on the possibility of being an active agent of contagion for their beloved ones, the basic emotion of fear should be added to the complex emotion of potential fuzzy guilt). So, if in-group/out-group dynamics—up to the attitudes toward the “stranger” in a geographical and political sense (Antonietti and Marchetti, 2020)—are the result of this primary articulation according to which “known-familiar” equals to reliable and “unknown-unfamiliar” equals to potentially dangerous (danger from which—phylogenetically and ontogenetically—the “known-familiar” is in charge of protecting us), the effect of the fuzziness of emotions, and especially of fear on mental health in a stressful situation like the one represented by the COVID-19 pandemic, can be easily imagined. In fact, the COVID-19 pandemic implies the possibility of indiscriminate contagion by anyone, including those closest to us in a psychic sense. Because of this, it undermines the dynamics depicted above by eliciting an unprecedented form of fear, in which the boundaries between safety and risk fall. If infected, it is necessary to adhere to the rule of indiscriminate social distancing from everyone. The same applies if a relative is infected. The work of mercy to “visit the sick” cannot be accomplished, just as it is impossible to extend the final farewell to those who left us forever. In a word, COVID-19 has completely changed the physiognomy of security/trust/danger/risk and fear, suddenly destroying a bond that evolution and ontogenetic development have taken a long time to build. The feelings of neglecting if not abandoning the beloved ones, or to be neglected if not abandoned by them to ensure the protective purposes of social distancing, are not easy to be managed from a psychological point of view; the experience of isolation, loneliness, and the worry of being forgotten are difficult to explain and to make comprehensible for children as well as the elderly. This is to say that the erosion of the foundations of the distinction between “known-familiar-safe/unknown-stranger-unsafe” could vary according to the developmental phases of the individual as well as the status of experts/novices. In terms of developmental phases, the cognitive, social, and affective resources typical of specific ages allow children to assimilate and elaborate differently information about the virus, its effects, and the dangers of proximity to beloved people. On the other end, if viewed from the perspective of expert/novices status, which is partially connected with the developmental phases, to have reliable information or real scientific knowledge on the spread of the virus could help to better manage the effect of the new form of fuzzy fear. Going back to the role played by robotics within the psychological framework briefly outlined here, the use of robots may change depending on a series of factors that only the contribution of psychologists may help to highlight. First of all, the “like me experience,” which represents the basis of acceptance/refusal of social robots, changes with age. Like the people's sense of people (to paraphrase Legerstee, 2005), also people's sense of social robots depends on the development, as well as the aims and contexts, of the robots' use (Marchetti et al., 2018). For these reasons, it is fundamental that the design of social robots meant to be deployed in situations of “fuzzy fear” like the one we are experiencing not only includes the purposes of assistance, companionship, or tutoring associated with medical regimens but also takes the real role of “fear-free” mediators of affective functions. In this way, robots do not become substitutes for close relationship partners from whom social distancing separates us, but act as relational bridges between those who are separated for health and safety reasons. As an effect of this rethinking the functions of social robots in emergency situations, some current negative attitudes toward social robots—from resistance and ambivalence up to the uncanny valley phenomenon (Mori, 1970; MacDorman and Ishiguro, 2006)—could significantly change. To pursue the goal of designing useful social robots for the psychological needs described here (i.e., coping with fuzzy fear and taking advantage of robots as affective mediators), a deep, psychologically driven afterthought will be needed around three basic axes of reflection. The first two axes are more general. The first one regards the psychological understanding of people involved in human–robot interactions during a sanitary emergency in terms of level of development, socio-demographic characteristics, and previous experience with social robots (see the experts/novices distinction above). Expectations and attitudes toward social robots may in fact change according to both development and expertise. The second axis regards the construction of social robots that are able not only to take into account the needs of their human partners but also to relate with the human agent in an understandable way. This represents an extremely important feature that every human would expect from the interactive experience. The literature on robotics calls it “transparency”/“explainability” (Holzinger et al., 2019), which would correspond to the experience of the Theory of Mind (Perner, 1991; Wellman et al., 2001) in the domain of human–human interaction. The third axis of reflection relates to a goal that we hope to achieve in a not too distant future. Specifically, it concerns the identification of the best way to devise social robots that are able to sensitively manage and respond to the behavior of a human partner with a possible acute temporary breakdown in the ability to scaffold the sense of emotional security—like some of us during this COVID-19 emergency—that is the very basis of Self construction.

The theoretical reflections discussed in this Opinion reread therefore the question of fear in the light of a danger that poses new questions and that, as is suggested, leads to rethinking particular psychological and social dynamics. In reading the new relational dynamics hypothesized in the present work, from which the robot is spared, COVID-19 pandemics added novelty to the physiognomy of fear, which (unlike anxiety) is an emotion linked to objects and situational antecedents, and which may therefore be affected by the nature of its objects at the level of subjective experiences, behavioral reactions, as well as coping strategies. These theoretical suggestions may enrich knowledge from an interdisciplinary perspective, such as robotics and psychology, providing important starting points for future research by emphasizing which psychological components should be investigated in people interacting with robots. An example is the perception of in-group/out-group, as well as the components of fear that, in our opinion, are mitigated toward robots in the specific COVID-19 situation, which forces us to adapt to the inclusion of new social agents devoted to care assistance.

## Author Contributions

All authors contributed to the writing of the manuscript.

## Conflict of Interest

The authors declare that the research was conducted in the absence of any commercial or financial relationships that could be construed as a potential conflict of interest.

## References

[B1] Ainsworth M. D. Blehar M. C. Waters E. Wall S. (1978). Patterns of Attachment: A Psychological Study of the Strange Situation. Hillsdale, NJ: Erlbaum.

[B2] Antonietti A. Marchetti A. (2020). Migrazioni e psicologie. Introduzione al forum. Ricerche Psicol. 43, 13–19. 10.3280/RIP2020-001002

[B3] Bowlby J. (1988). A Secure Base. New York, NY: Basic Books.

[B4] Di Dio C. Manzi F. Itakura S. Kanda T. Ishiguro H. Massaro D. (2019). It does not matter who you are: fairness in pre-schoolers interacting with human and robotic partners. Int. J. Soc. Robot. 1–15. 10.1007/s12369-019-00528-9

[B5] Di Dio C. Manzi F. Peretti G. Cangelosi A. Harris P. L. Massaro D.. (2020a). Shall I trust you? From child human-robot interaction to trusting relationships. Front. Psychol. 11:469. 10.3389/fpsyg.2020.00469 32317998 PMC7147504

[B6] Di Dio C. Manzi F. Peretti G. Cangelosi A. Harris P. L. Massaro D. (2020b). Come i bambini pensano alla mente del robot: il ruolo dell'attaccamento e della Teoria della Mente nell'attribuzione di stati mentali ad un agente robotico [How children think about the robot's mind. The role of attachment and Theory of Mind in the attribution of mental states to a robotic agent]. Sistemi Intellig. 1, 41–56. 10.1422/96279

[B7] Ekman P. Friesen W. V. (1971). Constants across cultures in the face and emotion. J. Pers. Soc. Psychol. 17:124. 5542557 10.1037/h0030377

[B8] Holzinger A. Langs G. Denk H. Zatloukal K. Müller H. (2019). Causability and explainability of artificial intelligence in medicine. Wiley Interdisc. Rev. Data Mining Knowl. Discov. 9:e1312. 10.1002/widm.1312 32089788 PMC7017860

[B9] Legerstee M. (2005). Infants' Sense of People: Precursors to a Theory of Mind. Cambridge: Cambridge University Press.

[B10] MacDorman K. F. Ishiguro H. (2006). The uncanny advantage of using androids in cognitive and social science research. Interact. Stud. 7, 297–337. 10.1075/is.7.3.03mac

[B11] Manzi F. Ishikawa M. Di Dio C. Itakura S. Kanda T. Ishiguro H.. (2020a). The understanding of congruent and incongruent referential gaze in 17-month-old infants: an eye-tracking study comparing human and robot. Sci. Rep. 10:11918. 10.1038/s41598-020-69140-6 32681110 PMC7368080

[B12] Manzi F. Peretti G. Di Dio C. Cangelosi A. Itakura S. Kanda T.. (2020b). A robot is not worth another: exploring children's mental state attribution to different humanoid robots. Front. Psychol. 10:2011. 10.3389/fpsyg.2020.02011 33101099 PMC7554578

[B13] Marchetti A. Manzi F. Itakura S. Massaro D. (2018). Theory of mind and humanoid robots from a lifespan perspective. Zeitschr. Psychol. 226, 98–109. 10.1027/2151-2604/a000326

[B14] Mori M. (1970). The uncanny valley. Energy 7, 33–35.

[B15] Perner J. (1991). Understanding the Representational Mind. Cambridge, MA: The MIT Press.

[B16] Pianta R. C. (1999). Enhancing Relationships between Children and Teachers. Washington, DC: American Psychological Association.

[B17] Schaffer H. R. (1966). The onset of fear of strangers and the incongruity hypothesis. J. Child Psychol. Psychiatry 7, 95–106. 10.1111/j.1469-7610.1966.tb02167.x 5969990

[B18] Spitz R. A. (1945). Hospitalism: an inquiry into the genesis of psychiatric conditions in early childhood. Psychoanal. Study Child. 1, 53–74. 10.1080/00797308.1945.11823126 21004303

[B19] Wellman H. M. Cross D. Watson J. (2001). Meta-analysis of theory-of-mind development: the truth about false belief. Child Dev. 72, 655–684. 10.1111/1467-8624.00304 11405571

[B20] Yang G. Z. Nelson B. J. Murphy R. R. Choset H. Christensen H. Collins S. H. (2020). Combating COVID-19—the role of robotics in managing public health and infectious diseases. Sci. Robot. 5:eabb5589 10.1126/scirobotics.abb5589 33022599

